# Fast Detection of Olive Trees Affected by Xylella Fastidiosa from UAVs Using Multispectral Imaging

**DOI:** 10.3390/s20174915

**Published:** 2020-08-31

**Authors:** Attilio Di Nisio, Francesco Adamo, Giuseppe Acciani, Filippo Attivissimo

**Affiliations:** Department of Electrical and Information Engineering, Polytechnic University of Bari, 70125 Bari, Italy; attilio.dinisio@poliba.it (A.D.N.); giuseppe.acciani@poliba.it (G.A.); filippo.attivissimo@poliba.it (F.A.)

**Keywords:** xylella fastidiosa, multi-spectral imaging, UAV, unmanned aerial vehicle, image processing, machine learning, precision agriculture

## Abstract

*Xylella fastidiosa* (*Xf*) is a well-known bacterial plant pathogen mainly transmitted by vector insects and is associated with serious diseases affecting a wide variety of plants, both wild and cultivated; it is known that over 350 plant species are prone to *Xf* attack. In olive trees, it causes olive quick decline syndrome (OQDS), which is currently a serious threat to the survival of hundreds of thousands of olive trees in the south of Italy and in other countries in the European Union. Controls and countermeasures are in place to limit the further spreading of the bacterium, but it is a tough war to fight mainly due to the invasiveness of the actions that can be taken against it. The most effective weapons against the spread of *Xf* infection in olive trees are the detection of its presence as early as possible and attacks to the development of its vector insects. In this paper, image processing of high-resolution visible and multispectral images acquired by a purposely equipped multirotor unmanned aerial vehicle (UAV) is proposed for fast detection of *Xf* symptoms in olive trees. Acquired images were processed using a new segmentation algorithm to recognize trees which were subsequently classified using linear discriminant analysis. Preliminary experimental results obtained by flying over olive groves in selected sites in the south of Italy are presented, demonstrating a mean Sørensen–Dice similarity coefficient of about 70% for segmentation, and 98% sensitivity and 93% precision for the classification of affected trees. The high similarity coefficient indicated that the segmentation algorithm was successful at isolating the regions of interest containing trees, while the high sensitivity and precision showed that OQDS can be detected with a low relative number of both false positives and false negatives.

## 1. Introduction

With a mean production of 450–550 $\times 10^{6}$ kg/year of olive oil, olive tree cultivation is undoubtedly one of the main sources of agricultural revenue for Italy. In particular, the Apulia region in the south, with over 360 kha (kilohectares) covered with 21 different olive cultivars with a prevalence of *Ogliarola* and *Coratina* cultivars, is the region with the highest percentage of production (>35% of the total yearly Italian production) [1]. In the past decade, this production has been greatly impacted by many threats, primarily *Xylella fastidiosa (Xf)*, a pathogen that has been known around the world for decades, but which since 2013 has put the survival of Apulian olive cultivation at great risk. It is a bacterium that can attack olive trees, vines, oleander, and some species of citrus fruits, causing them to rapidly dry out. This phenomenon, when observed on the olive trees, is known as olive quick decline syndrome (OQDS) [2,3,4,5,6,7,8,9,10,11].

*Xf* is endemic to the American continent and, until recently, it did not exist in Europe [12]; indeed, its arrival in Europe was tracked back to the import of some infected ornamental plants from Costa Rica (Central America) to Gallipoli (province of Lecce in southern Italy) in 2013 [13]. From there, the bacterium spread to the northwest provinces of Brindisi and Taranto, and some infected trees have also been recently reported in the province of Bari (northeast). A large number of publications about the impact of *Xf* in Puglia are available; a small selection is included in the references [14,15,16,17,18,19,20,21,22,23,24,25,26,27,28,29,30,31].

The main problems concerning the detection of this disease in olive trees are the possible lack of symptoms over an incubation period ranging from 6 to 18 months from infection and the nonuniform distribution of the bacterium on the infected plants, making it somewhat difficult to identify until it is too late.

Characterization of infection spread may potentially be achieved as was done previously for citrus using geostatistical analysis and kriging estimation, which might also be combined with Kalman filter prediction; however, the availability of data from extended monitoring is fundamental [32,33].

To date, the most accurate method to detect the presence of the *Xf* bacterium is by means of laboratory genetic analyses using the PCR technique (polymerase chain reaction [34,35]), a sophisticated and complex technique used to reproduce small segments of DNA many times in order to be able to process them in successive tests. The PCR technique is more sensitive than serological analyses of the ELISA type (enzyme-linked immunosorbent assay [36,37]), which are sensitive to antibodies or antigens of a given pathogen. Due to their inherent lower sensitivity, ELISA-type tests can produce a greater number of false negatives. However, both these techniques require medium to long waiting times (some days) to produce results and are applicable only in a laboratory using high-cost analytical instruments, so they are not applicable in real time in the field. Indeed, they require intensive in situ inspection though interesting methods useful to estimate water content and thermal characterization have been proposed [38,39,40,41]. Recently introduced alternative techniques involve proximity or remote sensing, i.e., the use of electromagnetic radiation and its interaction with objects and living beings. The advancement of satellite and aerial detection techniques, telecommunications systems and optical sensors has led to the application of these analysis techniques to images acquired by satellites (remote sensing by satellite), small manned planes or helicopters, or aerial platforms with a remote pilot (unmanned aerial vehicles; UAVs), commonly called “drones” in a wide range of fields [41,42,43,44,45]. UAV platforms can be further distinguished as fixed-wing types, which allow monitoring of large areas from medium–high altitudes, and rotary-wing types, which allow observation of less extensive areas from medium–low altitudes. In recent years, the application of UAVs in precision agriculture as well as in many other fields is becoming more and more common, requiring test systems able to guarantee and certify both electrical and mechanical performance aspects of their propulsion subsystems [46,47,48].

Typically, the radiation reflected by vegetation is concentrated in the visible (VIS), near-infrared (NIR), and medium infrared (SWIR; short-wave infrared) spectral regions, while the emitted radiation is concentrated in the thermal infrared (TIR) spectral region.

Spectral analysis finds frequent and extensive use in many areas of the physical sciences; this technique is one of the statistical methods used to characterize and analyze sequenced data in one-, two-, and three-dimensional space. In this area, many studies have been devoted to reducing bias and variance of the estimates [49,50].

The spectral signature of vegetation, which is the relative intensity of the re-radiated radiation as a function of the wavelength of the incident light, contains a range of information. Indeed, the shape of this curve

- depends on the photosynthetic activity in the VIS region;
- depends on the structure of plants’ leaves and foliage (size, number of leaf layers, etc.) in the NIR region;
- is strongly influenced by the water content in the SWIR region.

The use of terrestrial or aerial drones, both manned and unmanned, equipped with multi- or hyperspectral image cameras to study the health status of plantations of various kinds is not a novelty in precision agriculture or for forestry monitoring [51,52,53,54,55]. There are also pioneering applications of drones for the detection of *Xf*-infected plants [56,57,58,59].

*Xf* represents such a serious threat to the future survival of the olive orchards in Italy that the Italian Ministry of Economic Development (MiSE) recently committed €3.5 million to funding another important research project named REDoX (Remote Early Detection of Xylella), which is focused on the detection and monitoring of *Xf* using multispectral, hyperspectral, and thermal imagery obtained by aircrafts, UAVs, and satellites. The REDoX project is being coordinated by the Apulian Aerospace Technological District (DTA) [60].

In this paper, it is shown that multispectral imagery shot using a midsized rotary-wing UAV can be successfully used to evaluate the health of olive trees in nearly real time with respect to olive quick decline syndrome due to *Xf*. For this purpose, a tree segmentation algorithm was developed and linear discriminant analysis (LDA) was applied to multispectral stacks. In Section 2, after a brief introduction to remote sensing in agriculture, the equipment used in this research and standard vegetation indexes are described. The proposed algorithm is also presented: image preprocessing is described Section 2.1; 3D reconstruction of the scene is described in Section 2.2; tree segmentation is detailed in Section 2.3; and health status classification is described in Section 2.4. Experimental results and performance evaluation are provided in Section 3, followed by discussion in Section 4 and conclusions in Section 5.

## 2. Materials and Methods

**Figure 1:**
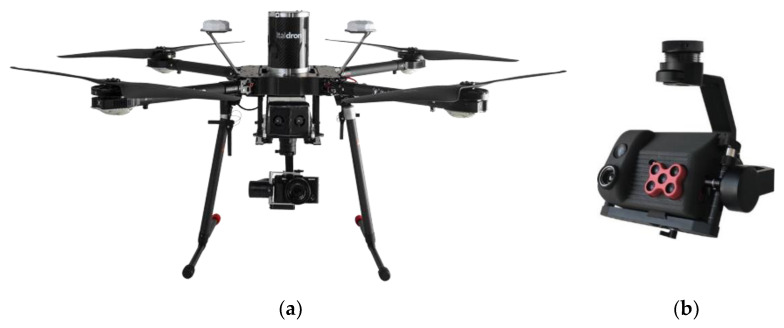
(**a**) The multirotor UAV (Unmanned Aerial Vehicle) used for this work; (**b**) its payload.

For the research work presented in this paper, which started almost two years ago and which was funded by the Apulia Region, a medium-sized multirotor UAV (Italdron 4HSE EVO, Figure 1a) with a maximum payload of 2.5 kg was used. It was equipped with a purposely developed payload comprising a 3D gimbal with a compact five band multispectral camera (MicaSense RedEdge-M [61]), a high-resolution thermal camera (FLIR Vue Pro 640 [62,63]), and a high-resolution visible camera (Sony α7r) (Figure 1b).

**Figure 2:**
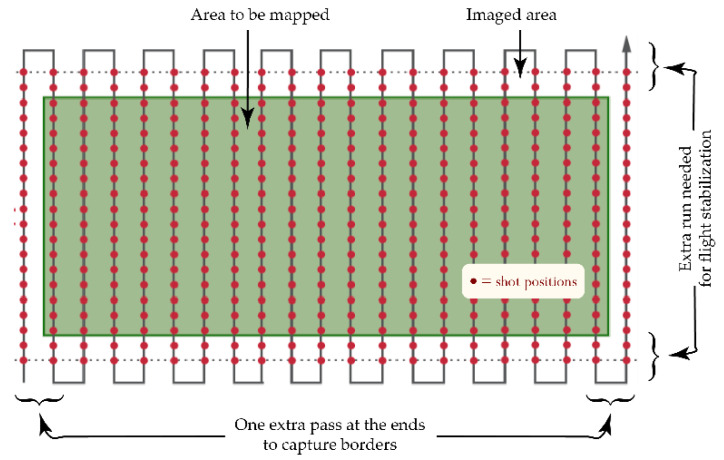
Typical UAV trajectory for an aerial survey (red dots identify shot positions).

This apparatus was used to acquire high-resolution images of olive groves from medium altitudes (some tens of meters). Surveys were done using the typical aerial-mapping “serpentine” profile (Figure 2) with high across- and along-track superposition percentages in order to collect enough data to be able to rebuild the whole high-resolution orchard image used in the processing step. Multiple surveys of the same olive grove were acquired from different altitudes to obtain different ground sample resolutions (GSD), to provide the opportunity to test the robustness and speed of the adopted processing solution. The above-ground levels (AGL) of the different surveys were calculated using a purposely developed spreadsheet, taking into account all inherent parameters: camera sensor resolution and focal length, desired ground sample resolution, camera shutter speed, UAV translation speed, etc.

**Figure 3:**
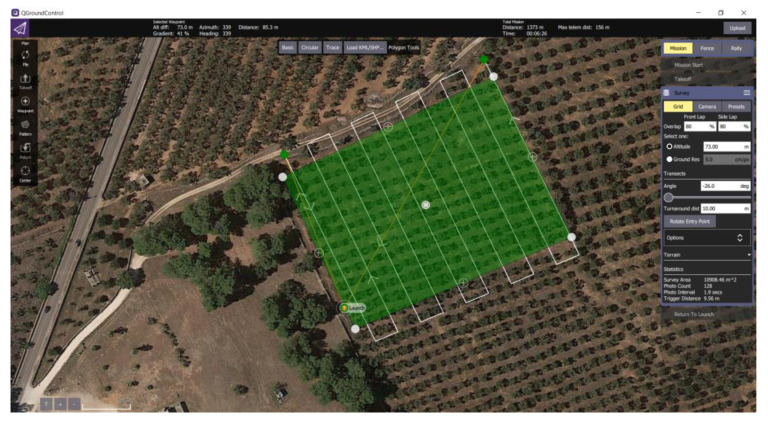
Example of one aerial survey plan for an olive orchard (Squinzano-Cerrate, 40°27′35.79″ N, 18° 7′2.69″ E).

Figure 3 shows a screenshot of the mission plan for the aerial survey on the Squinzano–Cerrate olive orchard.

**Figure 4:**
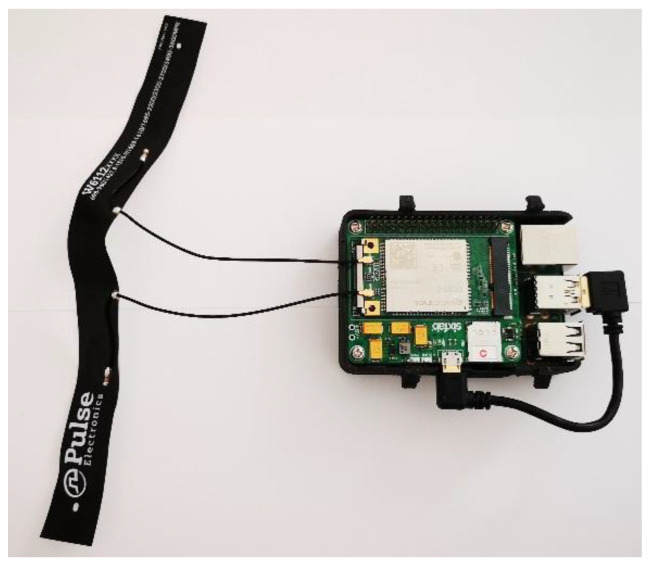
The Raspberry Pi SBC with the MiniPCI 4G/LTE Module used onboard the UAV to upload acquired images to the remote server.

The UAV used to acquire the aerial images was also equipped with an extra payload: a Raspberry Pi v4B + SBC (single-board computer) equipped with a 3G-4G/LTE Base Shield v2 by Sixfab GmbH plus an EC25 MiniPCI 4G/LTE Module by Quectel [64,65,66]. This was a nonstandard payload, the practical realization of which is shown in Figure 4, and it was purposely programmed and a customized 3D case was designed and printed to integrate it with the whole system. Thanks to this subsystem, the acquired images were transferred to a remote server where the developed software ran and where a GIS (geographic information system) platform completed the tagging and georeferencing of acquired images. Where the cellular network coverage on the field was good, the upload process was executed during the UAV’s flight; elsewhere, it was executed offline when the UAV landed. In fact, during field tests the cellular network’s availability and bandwidth represented a clear bottleneck for the real-time image upload process. However in the near future, when the coverage of new and higher-bandwidth cellular networks is sufficient, this problem should be resolved or at least mitigated.

The image-processing software developed for this work was written in Python 3.7 extended with many additional modules. It processed multispectral images related to a single overflight, performing the essential alignment operation of images returned by the five different sensors of the multispectral camera and those taken by thermal and visible camera, and returning multispectral and calibrated reflectometric stacks. Spectral characteristics of the MicaSense RedEdge-M sensors are given in Table 1.

Starting from these intermediate multispectral stacks, the software returned the following images in TIFF format:

- **RGB images** (red–green–blue), i.e., common visible images.
- **CIR images** (color and infrared = Red + Green + NIR) were obtained by substituting the NIR (near-infrared) images into the blue channel on the common RGB images. NIR wavelengths are effective in penetrating atmospheric mist and in determining the health of vegetation. The pigment in the leaves of plants, chlorophyll, strongly absorbs visible light, but the cellular structure of healthy leaves, on the other hand, strongly reflects NIR radiation. Therefore, the stronger the NIR radiation detected by the camera, the healthier the plant is.
- **NDVI images.** In normalized-difference vegetation index images, each pixel was calculated from the pixels of the same position in the NIR and RED images through the well-known relationship

(1) $$NDVI\;=\;\frac{NIR\;-\;RED}{NIR\;+\;RED}$$

The NDVI value is highly informative about the local health status of plants and soil, since it allows immediate recognition of areas of the canopy or of the underground soil that have development or irrigation problems. The interpretation of NDVI values is relatively simple; in fact, its value varies between 0 and 1, and each value corresponds to a different agronomic situation, regardless of the crop [67,68].

The NDVI value can be influenced by a variety of factors including soil brightness, air humidity, and plant foliage structure. Therefore, two NDVI maps taken on different days in the same field can look completely different due to different weather and/or light conditions. This can make the comparison of such NDVI images difficult, so it is essential to calibrate the remote-sensed images using a radiometric calibration panel on the ground before the acquisition process, or by other means. In our case, the multispectral Micasense RedEdge-M camera was equipped with its optional DLS (downwelling light sensor) module, a five band light sensor which is mounted on the top of the UAV pointing toward the sky and connected directly to the camera. The DLS module continuously measured the ambient light during the flight for each of the five bands of the camera and recorded this information in the metadata of the captured images.

The experimental data illustrated in Section 3 of this paper were acquired on 19 April 2019 at two sites located in Apulia, southern Italy, with a clear sky.

- *San Vito dei Normanni* (BR), 40°38′1.12″ N, 17°42′49.10″ E, time 12:45 UTC, with healthy olive trees with a planting layout of about 10 m × 12 m and an age of about 80 years. Sun elevation and azimuth were 51° and 229°, respectively.
- *Squinzano* (LE), 40°27′35.79″ N, 18° 7′2.69″ E, time 14:30 UTC, with olive trees with symptoms of *Xf*, with a planting layout of about 8 m × 8 m and an age of about 50 years. Sun elevation and azimuth were 33° and 255°, respectively.

Images were taken with the MicaSense RedEdge-M multispectral camera with 1.2 megapixel resolution, flying at an AGL (above-ground level) of 70 m; ground sample resolution was 5 cm/pixel, and forward/cross-overlaps during image acquisition were 80%. The acquired images were processed as described in following subsections and to develop and validate the machine-learning algorithms they were organized into a training and a test set, both containing 71 trees.

For the two critical tasks of the algorithm, namely the automatic tree segmentation and the tree classification, performance figures were evaluated. In particular, for tree segmentation, it was evaluated whether the binary mask representing trees overlapped well with actual trees. Thus, the Sørensen–Dice similarity coefficient (DSC) was calculated for each tree (trees with merging foliage were treated as a single tree), and its statistics were reported. This similarity coefficient represents the degree of matching between two binary images, where each pixel in common contributes to increasing the coefficient towards the limiting value of 1 (perfect match) and each different pixel decreases the coefficient towards the limit of 0 (complete absence of matching pixels). The DSC was calculated as twice the number of pixels common to the two sets obtained via automatic segmentation (proposed algorithm) and manual segmentation (ground truth), respectively, divided by the sum of the number of pixels in each set. The ground truth was obtained by manually segmenting RGB and CIR images extracted from the multispectral stacks. A few trees that were not olive trees were individuated and excluded in the evaluation of segmentation and classification performance.

The confusion matrix, sensitivity, and precision of the tree classification were calculated. The ground truth of health status was assessed by an expert agronomist and was compatible with information about the Apulia area provided by the institutional Apulia monitoring system for *Xf* [69].

Further details and results are provided in Section 3.

**Figure 5:**
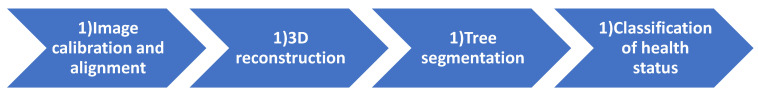
Main processing tasks of the proposed system.

Data processing in the proposed system was subdivided into four main tasks, which are listed in Figure 5. They are analyzed in the following subsections. The list of main mathematical symbols can be found in Table 2.

### 2.1. Image Calibration and Alignment

**Figure 6:**
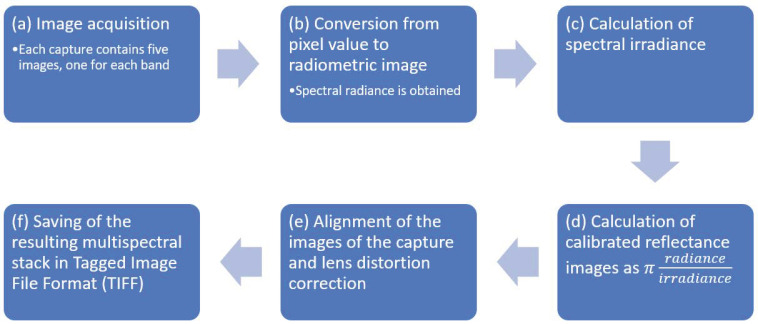
Steps of image calibration and alignment.

The five images grabbed from the multispectral sensor were calibrated and aligned as discussed below to obtain a proper multispectral reflectance stack, following the procedure recommended by the manufacturer [70]. The processing steps are summarized in Figure 6 and described below.

- Input data were constituted by shots, also named “captures”, where each capture contained five raw digital images in Tagged Image File Format (TIFF), acquired synchronously by the sensors of the five band multispectral camera. One capture was processed at a time.
- The spectral radiance in wavelength, units $W\cdot m^{-2}\cdot {\mathrm{sr}}^{-1}\cdot {\mathrm{nm}}^{-1}$, was obtained from pixel values in each raw image, taking into account calibration and lens vignette effect parameters provided by the manufacturer, as well as exposure time, black level, and gain of the imaging sensors at the time the images were shot. Spectral radiance images $L_{i}$, i=1,…,5 were obtained, where each $L_{i}$ is an array.
- Spectral irradiances, $E_{1},\ldots E_{5}$, which are the amount of energy per unit area per unit bandwidth ($W\cdot m^{-2}\cdot {\mathrm{nm}}^{-1}$) incident on the ground, were calculated from data measured by the downwelling light sensor (DLS) mounted on the drone. Data were acquired by the DLS at the same time as the images were captured by the multispectral camera. When calculating irradiance, the position of the DLS (measured by an onboard sensor) and solar orientation were considered, and clear sky conditions were assumed.
- Spectral reflectance images $R_{i}$ were obtained from the ratio of reflected and incident light, calculated precisely as
  (2) $$R_{i}\;=\;\pi \frac{L_{i}}{E_{i}}\;$$
- Reflectance images were corrected for lens distortion using parameters provided by the manufacturer, namely three-element radial distortion and two-element tangential distortion correction parameters. After distortion correction, the five images were aligned by correcting the different points of view of each sensor. For this purpose, the image in the green band was (arbitrarily) selected as a reference, and the other four images were aligned by calculating, for each of them, an eight-parameter homography that maximized the enhanced correlation coefficient (ECC) [71] with the reference. The parameters were obtained using the *findTransformECC* function of the OpenCV library [72]. Since estimating the parameters of the four homographies was time-consuming, they were calculated for a capture in the middle of the flight and their inverse functions were applied to align the other captures; this procedure was correct because all of them were taken at the same distance from the ground, and at a distance which was greater than the change of depth of the subjects (trees and ground); hence, the images could be aligned in the same way.
- As a result, a properly aligned stack of spectral reflectances was obtained and saved in a five channel TIFF file. Channels in the stack were named BLUE, GREEN, RED, NIR, REDEDGE. Each channel represented an array of size $N_{r}\times N_{c}$, for example NIR(r,c) was the near-infrared reflectance of the pixel in row index r and column index c, where $r\;=\;1,\ldots ,N_{r}$ and $c\;=\;1,\ldots ,N_{c}$.

### 2.2. 3D Reconstruction

Images shot during the flight, which were taken from different perspectives as the drone moved, were merged using photogrammetric techniques to create a complete map of the site. The main outcome of this processing task, however, was the estimation of elevation of points in the images, which permitted improved identification of trees by separating them from the ground and grass.

It is well known that GPS information is only sufficient for a coarse mosaicing of images. What was needed here was, instead, an accurate mosaicing and alignment that could be obtained by individuating corresponding points on the partially overlapping margins of the images. For this reason, it was important that images were shot with high overlap. Moreover, if georeferencing is required with accuracy greater than that of GPS systems, ground trust points can be used; however, this was not deemed necessary for the purposes of the present study, since only relative height measurements were used, as is made clear in Section 2.3. Indeed, some advanced studies have been done to improve the accuracy of UAVs’ positioning with GPS [73].

In this work, the VisualFSM application was used, which allowed the 3D reconstruction using structure from motion [74]. Hence, as a result of this photogrammetric dataflow, a 3D reconstruction of the soil, vegetation, and buildings and an N-View Match (NVM) file were obtained. In particular, the latter file contained the 3D coordinates $\left(x_{i},y_{i},z_{i}\right)$ associated with the pixel index $\left(u_{i},v_{i}\right)$ of matched points between the images that were used in the subsequent segmentation technique. The set of K matched points for a given image was denoted as $P=\left\{\left(u_{i},v_{i}\right),\ldots ,\left(u_{k},v_{k}\right)\right\}.$

### 2.3. Tree Segmentation

**Figure 7:**
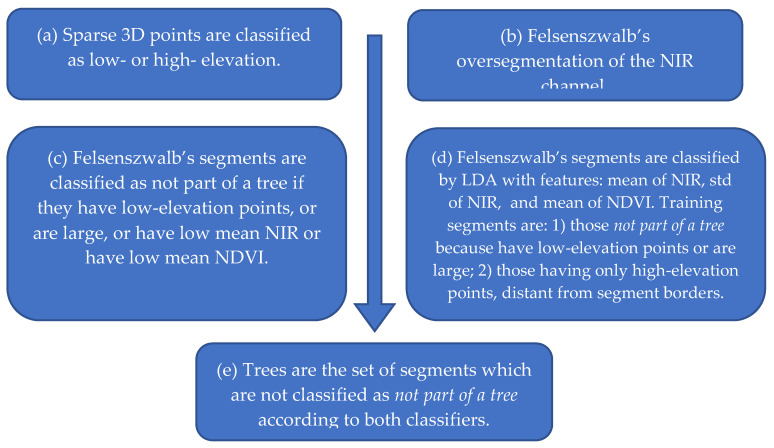
Steps of the tree segmentation algorithm.

Tree segmentation was performed according to the algorithm outlined in Figure 7, which is detailed below. Its purpose was to separate trees from soil and apply health status classification to trees only. The algorithm was composed of many steps because differentiating trees and their irregular contours from soil or even from soil covered with grass proved to be a difficult task.

(a) *Classification of Sparse 3D Points*

In the first step (a), several points were subdivided into low- and high-elevation to facilitate tree segmentation. Let $\left(x_{i},y_{i},z_{i}\right)$ be the 3D coordinates of a pixel $\left(u_{i},v_{i}\right)$, in the multispectral stack, where pixels $\left(u_{i},v_{i}\right)$ belong to the set of the K matched points P defined previously. Three-dimensional points are interpolated by a plan ${\tilde{z}}_{i}\;=\;\alpha x_{i}\;+\;\beta y_{i}\;+\;\gamma$, where α,β, and γ are obtained by the ordinary least-squares method. This plan represents a raw linear approximation of the ground surface. Afterwards, the residuals ${\tilde{z}}_{i}\;-\;\left(\alpha x_{i}\;+\;\beta y_{i}\;+\;\gamma \right)$ are calculated and standardized by subtracting their mean and dividing by their standard deviation to obtain the standardized residuals $e_{i}^{\prime }$. A point $\left(x_{i},y_{i},z_{i}\right)$ and its corresponding pixel $\left(u_{i},v_{i}\right)$ are classified as high-elevation if $e_{i}^{\prime }\;\geq \;t_{H}^{\prime }$, where $t_{H}^{\prime }$ is a purposely defined standardized elevation threshold.

(b) *Felsenwalb’s Oversegmentation*

In the second step (b), an oversegmentation is performed using Felsenszwalb’s method [75] applied to the NIR channel to obtain $N_{F}$ segments described by the pixel sets $F_{j}$, $j=1,\ldots ,N_{F}$. The *scikit-image* Python image-processing library is used for that purpose [76]. Parameters of the oversegmentation are scale $s_{F}$, standard deviation $\sigma_{F}$ of the Gaussian kernel for image preprocessing, and minimum component size $m_{F}$ enforced in postprocessing.

The purpose of this step is to obtain areas with contours that can follow the irregular borders of crowns of trees, for which the difference with soil is shown by the greater NIR reflectance. As a side effect, single trees are also oversegmented and split into many segments, which should be subsequently identified and merged as described later.

Afterwards, two different classification methods, in steps (c) and (d), are applied to Felsenszwalb’s segments, and their results are combined in step (e). Let $S_{j}^{\prime }$ and $S_{j}^{\prime \prime }$ be the classification results for the $F_{j}$ segment according to the first and second method, respectively, and let $S_{j}$ be the final classification into class $C_{NT}$ (“not part of a tree”) or class $C_{T}$ (“part of a tree”). We used, for example, notation $S_{j}=C_{NT}$ to say that segment $F_{j}$ has been classified as not part of a tree.

(c) *First Classification Method for Felsenszwalb’s Segments*

In the first method, step (c), a segment $F_{j}$ is classified into class $C_{NT}$ if the segment contains points on the ground or nonvegetation, otherwise it is classified into class $C_{T}$. More precisely, to classify a segment $F_{j}$ into class $S_{j}^{\prime }=C_{NT}$, at least one of the following four conditions should be satisfied.

(1) It contains at least one matched pixel below the elevation threshold. This is justified by the fact that if a segment contains low-elevation points, it is likely it belongs to the ground rather than trees. This condition is expressed as
  (3) $$\exists \;i\;s.t.\;\left(u_{i},v_{i}\right)\in F_{j}\;\wedge \;e_{i}^{\prime }<t_{H}^{\prime }\;$$
(2) Its area, i.e., the number of pixels, $a_{j}:\;=\;\#F_{j}$, divided by whole image area $N_{u}\times N_{v}$, is larger than the relative threshold $t_{area}^{r}$. This is justified by the fact that it is unlikely that large segments are part of trees. The relevant condition is
  (4) $$\frac{a_{j}}{N_{u}\times N_{v}}\;\geq \;t_{area}^{r}$$
(3) Its mean NIR reflectance is below the threshold $t_{NIR}$. Indeed, a low NIR reflectance can be associated with nonvegetation segments. This is expressed as
  (5) $$\frac{1}{a_{j}}\underset{\left(r,c\right)\in F_{j}}{\sum }NIR\left(r,c\right)\;\leq \;t_{NIR}$$
(4) Its mean NDVI is below the threshold $t_{NDVI}$. Analogously to NIR, a low NDVI can be associated with non-vegetation segments. This is expressed as
  (6) $$\frac{1}{a_{j}}\underset{\left(r,c\right)\in F_{j}}{\sum }NDVI\left(r,c\right)\;\leq \;t_{NDVI}$$
  where NDVI(r,c): = (NIR(r,c) − RED(r,c))/(NIR(r,c) + RED(r,c)).

If $F_{j}$ is not assigned to class $C_{NT}$, it is assigned by exclusion to class $C_{T}$, that is

(7) $$S_{j}^{\prime }\;=\;\{\begin{array}{c} C_{NT},\;\mathrm{if}\;\mathrm{any}\;\mathrm{of}\;\left(3\right),\left(4\right),\left(5\right),\left(6\right)\;\mathrm{is}\;\mathrm{satisfied}\\ C_{T},\;\mathrm{otherwise} \end{array}$$

(d) Second Classification Method for Felsenszwalb’s Segments

In the second segment-classification method, step (d), segments of the same image that can be classified more easily were used to classify the remaining segments via a machine-learning technique. Each segment was first assigned to class $C_{NT}$ if it contained low-elevation points or is large; to class $C_{T}$ if it contained only high-elevation pixels, distant from the borders of segments; or to class $C_{U}$ *(*“unknown”) otherwise. Afterwards, segments in class $C_{U}$ were reclassified using linear discriminant analysis (LDA) trained with segments already classified as $C_{NT}$ and $C_{T}$ on features that are statistics of NIR and NDVI. In detail, the algorithm of the second classification method is as follows.

(1) The training set of segments $F_{j}$ assigned to class $C_{NT}$, that is $S_{j}^{\prime \prime }\;=\;C_{NT}$, is built with any segment that contains at least one matched pixel below the elevation threshold or that is large—in other words, any segment that satisfies the previously defined condition (3) or the condition (4). For these segments, the condition to satisfy is
  (8) $$\exists \;i\;s.t.\;\left(u_{i},v_{i}\right)\in F_{j}\wedge \left(e_{i}^{\prime }\;<\;t_{H}^{\prime }\right)\vee \left(\frac{a_{j}}{N_{u}\times N_{v}}\;\geq \;t_{area}^{r}\right)$$
(2) The training set of segments of class $C_{T}$, for which $S_{j}^{\prime \prime }\;=\;C_{T}$, is built with any segment that satisfies both of the following conditions:
  - It has not been already classified into the training set of class $C_{NT}$, that is, both conditions (3) and (4) are not satisfied.
  - At least one high-elevation matched point of the segment is not on the border of all segments. Here, the border B of all segments is defined as the set of any pixel not completely surrounded (considering four-neighborhood connectivity) by pixels of the same segment, further thickened with an additional morphological binary dilation. This condition can be expressed as
    (9) $$\exists \;i\;s.t.\;\left(\left(u_{i},v_{i}\right)\in F_{j}\right)\wedge \left(e_{i}^{\prime }\geq t_{H}^{\prime }\right)\wedge \left(\left(u_{i},v_{i}\right)\notin B\right)\;$$
(3) Three features are calculated on each segment in the training set: arithmetic mean of NIR values over the pixels of the segment; standard deviation of NIR; arithmetic mean of NDVI. These features are used to train the LDA classifier.
(4) The trained LDA classifier is used to obtain the probability $p_{T,j\;}$ of each segments of class $C_{U}$ being in class $C_{T}$. Reassignment to class $C_{T}$ is performed if that probability is above the threshold $t_{T}$, otherwise the assignment is made to class $C_{NT}$.
  (10) $$S_{j}^{\prime \prime }=\{\begin{array}{c} C_{NT},\;\mathrm{otherwise}\\ C_{T},\;\mathrm{if}\;p_{T,j\;}>t_{T} \end{array}$$

(e) *Final Classification of Felsenszwalb’s Segments*

Final classification $S_{j}$ of each segment $F_{j}$ was performed in step (e) as follows. A segment was put in class $C_{T}$ (“part of a tree”) only if both classifiers agreed:

(11) $$S_{j}=\{\begin{array}{c} C_{NT},\;\mathrm{otherwise}\\ C_{T},\;\mathrm{if}\;S_{j}^{\prime }\;=\;C_{T}\wedge S_{j}^{\prime \prime }\;=\;C_{T} \end{array}$$

All the pixels that belonged to segments in class $C_{T}$ constituted the segmentation mask. In fact, the mask was subject to morphological binary erosion with a disk of radius $r_{e}$ to eliminate pixels at the border because there the crown density was lower and soil was partially visible. The final segmentation image L was obtained by labeling the connected components of the mask, where each component represented a tree or multiple trees touching each other.

### 2.4. Classification of Health Status

The last processing stage was the classification of olive trees in two classes according to their health status.

We investigated the feasibility of an approach in which the analysis was not limited to a few vegetation indices, but included all the spectral information across five bands.

In the proposed method, linear discriminant analysis (LDA) was used, which has already proven useful in medical applications for the assessment of diseases and for biological classifications.

Two classes of trees were defined: the class $C_{0}$ of negative trees, which were in good health, and the class $C_{1}$ of positive trees, which were in poor health. Analogously, two classes of pixels were defined: class $X_{0}$, which belonged to negative trees, and class $X_{1}$, which belonged to positive trees.

Briefly, in this paper, the LDA classifier was used to obtain a probability map p(r,c) that a pixel of the multispectral stack of coordinates (r,c) belonged to class $X_{1}$ of pixels of positive trees. If a tree contained a significant number of high-probability pixels, it was classified as positive (class $C_{1}$).

In further detail, in the training phase, trees were manually segmented and classified as $C_{0}$ and $C_{1}$, and the two sets of pixels of class $X_{0}$ and $X_{1}$ were used to train the LDA classifier. Each pixel value was a quintuple of reflectance values.

After training the classifier, it was used as follows on a new multispectral stack. In the first step, it operated at the pixel level, giving the already mentioned probability p(r,c). Indeed, the posterior probability p(r,c) of a pixel belonging to a class $X_{0}$ or $X_{1}$ was provided directly by the LDA classification technique [77]. Afterwards, each segmented tree was classified in the positive class if the average of its N higher-probability pixels was above the threshold t. Hence, given a segmented tree consisting of the set $T\;=\;\left\{\left(r_{1},c_{1}\right),\ldots ,\left(r_{M},c_{M}\right)\right\}$ of M pixels, the set of probabilities $P\;=\left\{p\left(r_{1},c_{1}\right),\ldots ,p\left(r_{M},c_{M}\right)\right\}$ was calculated by using the LDA classifier, and P was then ordered from maximum to minimum, giving the sequence $\left[p_{\left(1\right)},\ldots p_{\left(M\right)}\right]$. Finally, the mean value over only N pixels was calculated [78],

(12) $$v\;=\frac{p_{\left(1\right)}\;+\;\ldots +\;p_{\left(N\right)}}{N}$$

and the tree was classified into class C as follows.

(13) $$C\;=\{\begin{array}{c} C_{0},\;\mathrm{if}\;v<t\\ C_{1},\;\mathrm{if}\;v\geq t \end{array}$$

In this paper, the LDA implementation of the scikit-learn v0.20.3 Python package was used [79].

## 3. Results

In this section, results obtained by the data processing described in Section 2 are illustrated.

**Figure 8:**
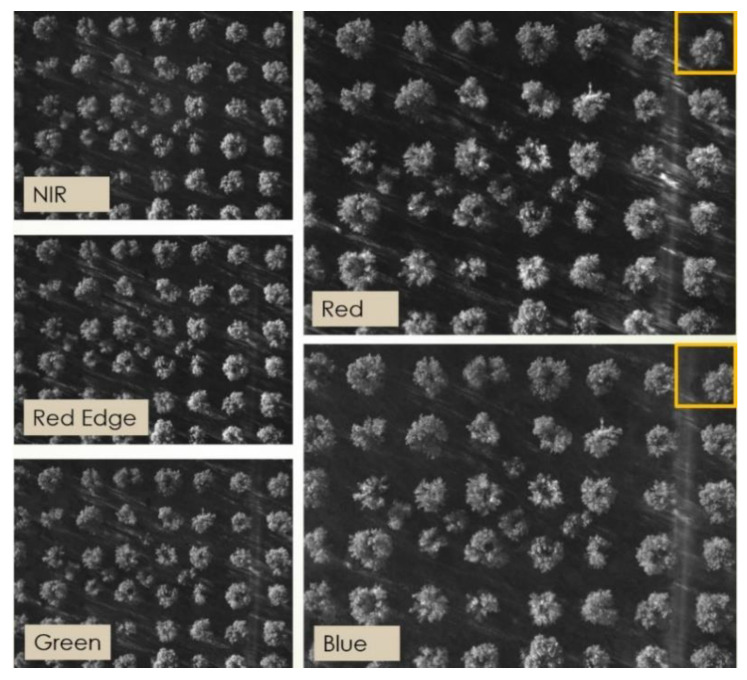
Example images of a multispectral stack. Each image is 960 pixels × 1280 pixels, corresponding to a ground size of about 64 m × 48 m.

**Figure 9:**
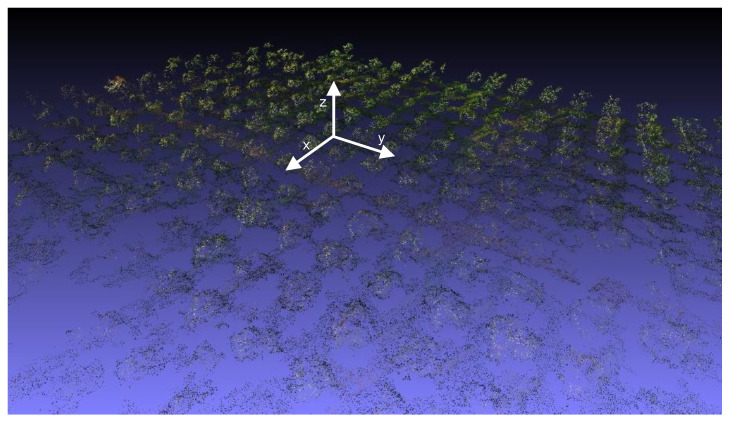
3D point cloud for the site in Squinzano.

**Figure 10:**
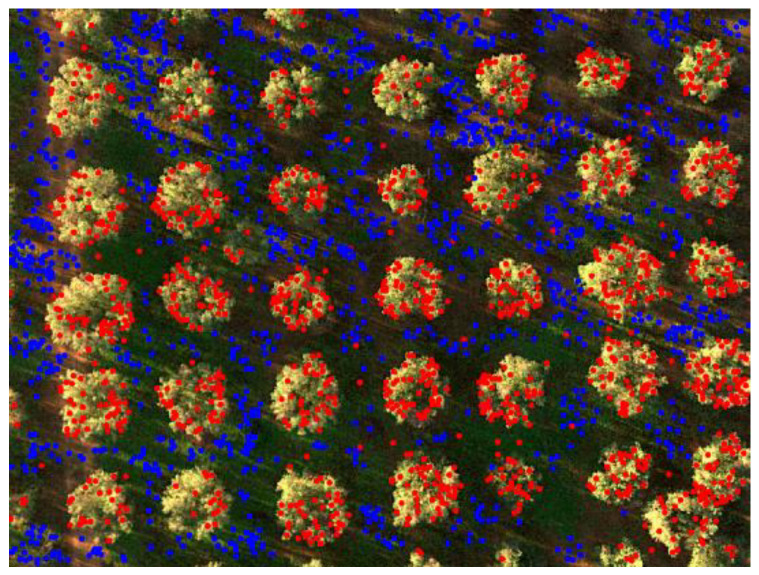
Points of the 3D cloud subdivided between (blue) ground and (red) tree crowns.

One example capture, before any processing, is shown in Figure 8, where the different points of view of, for instance, the red and the blue channels are apparent if one considers the tree indicated by the orange box in the upper right corner. All the acquired images were processed as described in Section 2.1 to obtain properly aligned multispectral reflectance stacks. Photogrammetric reconstruction was then applied to obtain the 3D point cloud shown in Figure 9 for the site in Squinzano. That reconstruction allowed us to distinguish between points on the ground and points on tree crowns, as shown in Figure 10, relevant to the site in Squinzano. Figure 10 is a single shot taken from an altitude of 70 m, has a size of 930 pixels × 1232 pixels or about 1.1 megapixels, and corresponds to a ground size of about 47 m × 62 m, with a resolution of 5 cm/pixel.

In the following part of the paper, results of the training phase are reported, followed by those relevant to the test phase. The latter were used to validate the proposed approach.

For training, the parameters for tree segmentation and classification, shown in Table 2, were found heuristically by considering one multispectral stack from each site, including 71 olive trees overall.

**Figure 11:**
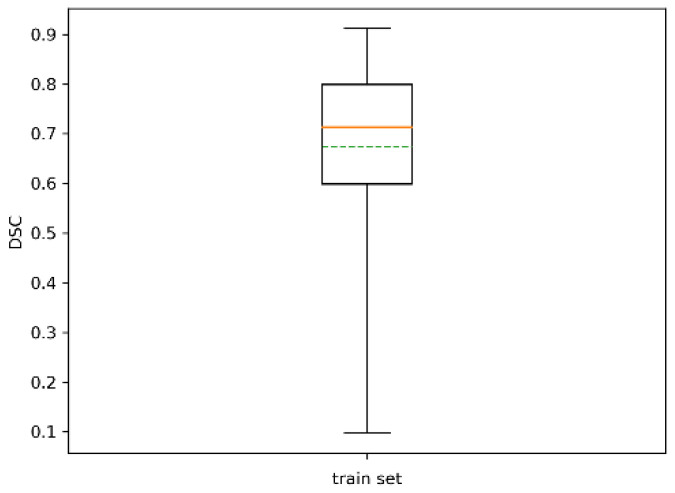
Boxplot of DSC for the training set. The dotted green line represents the mean.

The statistics of the resulting DSC, which expressed the Sørensen–Dice similarity coefficient between automatic segmentation and ground truth, are reported in Table 3 and Figure 11. The similarity coefficient averaged over all the trees was 0.68 with std 0.16, and its minimum was 0.12. All trees were identified.

The LDA classifier of plant health was trained using the previously described set of multispectral stacks. Only the pixels belonging to trees, as identified by the ground truth segmentation, were considered for training.

To evaluate the effectiveness of the training, the classifier was applied to the training set. It was used to obtain a probability map as described in Section 2.4. In particular, for each true tree area, the segmented regions which had at least one pixel in common with that area were selected and the probability map was evaluated. If the mean of the N = 2 higher-probability pixels was above the threshold t = 0.8, then that region was classified as positive. The resulting confusion matrix of the classifier is shown in Table 4, where four false-positive and zero false-negative can be observed. False positives were due to the inclusion of bare soil between branches of trees.

Hence the classifier sensitivity on the training set was 100%, and its precision was 93%.

After training, segmentation and classification performance were evaluated on test images taken on each site, including 71 olive trees overall. The automatic segmentation and classification were applied to each image to classify each tree.

**Figure 12:**
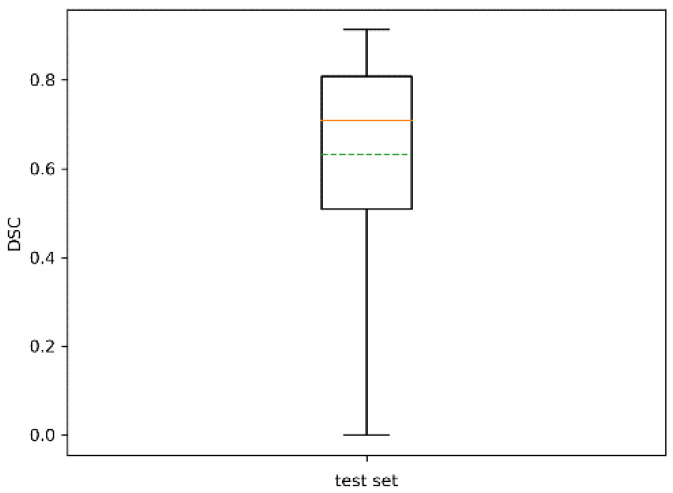
Boxplot of DSC for the test set. The dotted green line represents the mean. The dotted green line represents the mean.

The statistics of the Sørensen–Dice similarity coefficient are reported in Table 5 and in the boxplot of Figure 12. Its mean value was 0.66 with std 0.21. Performance decreased slightly with respect to the training set. However, it should be noted that all trees were successfully identified, at least on a portion of them, with a minimum DSC of 0.02.

Table 6 shows the confusion matrix for the test set. The observed classification accuracy was good, in agreement with previous results for the training set or even better, with 98% sensitivity and 100% precision.

Processing one image took 6.2 s for segmentation and 0.2 s for classification on a laptop with an Intel i7-8850H processor and 16 GB of RAM.

**Figure 13:**
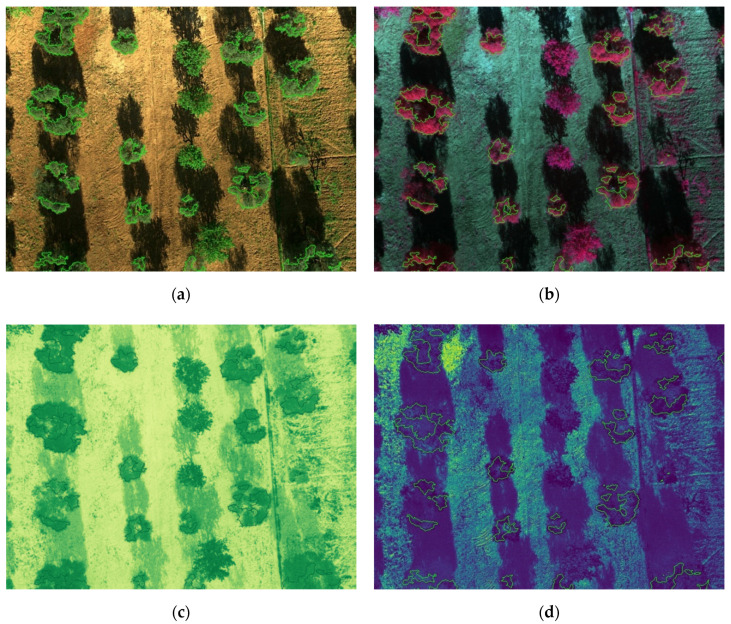
(**a**) Segmented RGB image; (**b**) segmented CIR image; (**c**) NDVI image; (**d**) probability map. A few trees that were not olive trees were excluded from processing.

**Figure 14:**
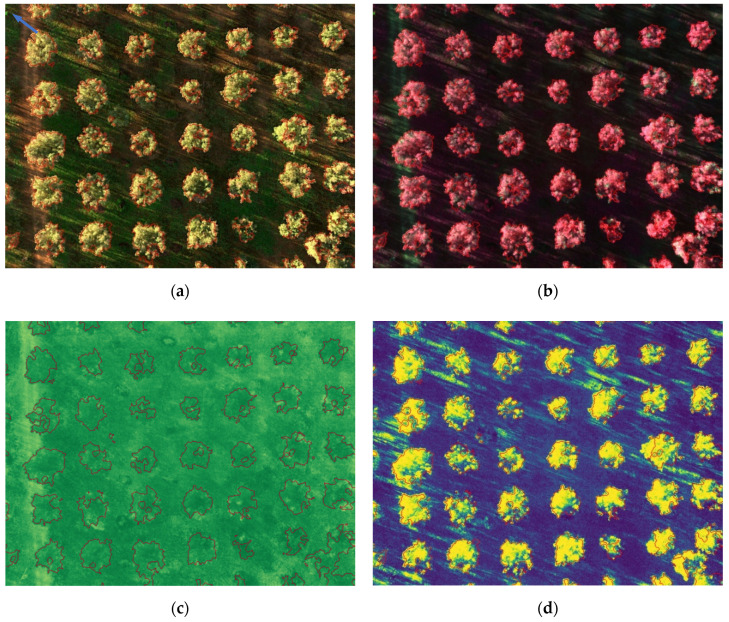
(**a**) Segmented RGB image; (**b**) segmented CIR image; (**c**) NDVI image; (**d**) probability map. The blue arrow in the upper left corner of (**a**) indicates a classification error.

Figure 13 and Figure 14 show the results for the test set, where segmented trees are surrounded with different colors, green for healthy ones or red for infected ones, depending on their health status as inferred by the algorithm. The images shown in Figure 13 were acquired at the San Vito dei Normanni olive grove, where olive trees were not affected by *Xf*. Figure 13d and Figure 14d show the probability map obtained by the classifier, represented as a blue to yellow color map, and the segmentation contour. The RGB, CIR, and NDVI images are also shown as an aid for understanding olive condition. Images of Figure 14 were acquired in Squinzano, where the olive trees were affected by *Xf* and clearly showed OQDS symptoms. These images are discussed in the following.

## 4. Discussion

The results shown in the previous section indicate that high classifier sensitivity and precision were achieved in both training and test sets.

It is worth noting that the test images were acquired in suboptimal conditions, with evident shadows cast on the ground and self-shadowing; however, both the segmentation and classification algorithms succeeded. This is very important, because due to logistic constraints, it is not always possible to survey a field under optimal irradiation, i.e., when the sun is directly overhead. Parameters such as gain and exposure time were optimized automatically by the multispectral camera according to shooting conditions and were saved in image metadata. According to manufacturer’s data, automatic gain control keeps the exposure below 2 ms. No attempt was made to correct specular reflection from vegetation, since it was considered negligible in the case of trees observed at that distance.

Altitude and resolution were such that individual leaves were not discernible; however, that level of detail was not deemed necessary and the analysis was performed on the spectral content of the crown as a whole. Moreover, lowering the altitude would have increased the time necessary for the survey, reaching the autonomy limit of the drone, while air moved by propellers during close-up shots would have shaken branches. Leaves’ and branches’ fluttering due to wind was low (7–9 mph wind from NNW) during the survey; however, it should be noted that flutter, if significant, may reduce the accuracy and reliability of photogrammetry.

Further details on the achieved result are discussed with the help of Figure 13 and Figure 14.

For the test set, only one misclassification error (false positive) was observed. It should be noted that the false negative was due to a very small branch, indicated by a blue arrow in the upper left corner of Figure 14a; given the small number of pixels, 24, it was not possible to detect that the tree was affected by *Xf*.

The figures illustrate the usefulness of considering all the spectral information, as was done by using the LDA classifier. Since the IR channel is represented with a false red color in CIR images, areas of a tree marked in green had smaller IR reflectivity, which is indicative of desiccation. This distinction becomes clearer if one compares Figure 13b (trees unaffected by *Xf*) and Figure 14b (trees affected by *Xf*), where desiccated parts are shown in green. However it is well known that IR reflectivity alone is not sufficient to evaluate the state of health of vegetation, and indices such as NDVI, which combines NIR and RED into a single value, have been widely adopted for this purpose [68]. Unfortunately, while NDVI is effective in differentiating healthy trees from bare soil, it provides no hints for distinguishing affected trees from healthy grass, as can be seen in Figure 13c. This motivated the usage of a suitable machine-learning algorithm based on at least the two-dimensional space constituted by NIR and RED values. It turned out that the usage of all the five bands was feasible and the proposed classification approach, based on the probability maps shown in Figure 13d and Figure 14d clearly separated affected tress from healthy trees, and from grass and bare soil.

It should be mentioned that recent studies on *Xf* have been conducted using hyperspectral sensors. The advantage of using hyperspectral indices is that precise diagnoses can be performed, distinguishing further between positive asymptomatic and symptomatic trees. This was used in Reference [67], where the phaeophytinization index (NPQI) calculated using narrow blue 415 and \5 nm spectral reflectance bands proved effective. In the same study, it was shown that NDVI did not differ significantly between asymptomatic and symptomatic trees. In our work, too, indices such as NDVI were discarded for the purpose of classification; however, we found that using multispectral imagery, which can be obtained with less demanding hardware than hyperspectral images, and directly processing the five broad bands with LDA was sufficient to discriminate between negative and symptomatic trees.

The same observation made for Figure 14c, that NDVI does not differentiate trees from grass, also motivated the approach used for tree segmentation. Indeed, the algorithm proposed in this paper takes into account several parameters, not only spectral ones but also points’ elevation, to accomplish the segmentation task in a robust way. However, it should be stressed that the elevation of points was obtained using photogrammetry on images shot with the multispectral sensor. In contrast, sensors such as LiDAR (light detection and ranging) can be used to ensure higher reconstruction accuracy and high point density, with good performance in the precise determination of canopy geometry, as was analyzed in Reference [79]. In our work, the precise analysis of canopy geometry was unnecessary and the obtained sparse 3D reconstruction, even if more prone to errors than LiDAR, nonetheless permitted identification of all trees thanks to our purposely developed segmentation algorithm.

As previously stated, a few non-olive trees were discarded from processing in Figure 13. That exclusion was performed manually. Automatic methods for tree species recognition have been proposed in the literature; LiDAR data together with geometric models were used to distinguish among five species in forest plots in Finland with a classification accuracy above 93% [80]. However, such a technique was not applied in our work in order to relax hardware requirements. In Reference [81], it was shown that shape, texture, and color analysis of airborne winter imagery taken with a pocket camera could discriminate between three boreal forest species with 82% accuracy. Even if tree segmentation was facilitated by snow on the ground in that study, it is foreseen that this class of techniques may be exploited to complement the method proposed in this paper, which does not suffer from background discrimination, in order to automatically select olive trees.

## 5. Conclusions

In this paper, a technique is presented to monitor the spread of olive quick decline syndrome (OQDS) in olive trees using remote sensing with a multispectral camera mounted on a multirotor UAV. The entire data flow is described, including preprocessing to obtain calibrated reflectance images, 3D reconstruction of a sparse cloud of points with stereophotogrammetry, segmentation of trees, and classification of their health status.

Segmentation was complicated by the presence of grass on the soil; hence, a hybrid approach was used based on the combination of multispectral information and spatial data, producing a mean Sørensen–Dice similarity coefficient of about 70% with respect to the ground truth. That value was worsened with respect to the intermediate segmentation result because it took into account the final morphological erosion of segmented trees aimed at reducing the superposition of terminal parts of branches with soil; that erosion was introduced because including soil areas may reduce classification accuracy. It can therefore be assumed that there is a trade-off between segmentation and classification accuracy. It should be appreciated that in one step of the segmentation algorithm, an LDA classifier was trained using decisions made on easy cases and applied to dubious ones. This technique has the advantage of being potentially self-adapting to different kinds of trees and soil. In this work, the LDA classifier was trained on each multispectral stack separately, but training on a whole set of homogenous stacks would increase further its reliability.

Detection of disease was based, again, on an LDA classifier trained on segmented trees from five band multispectral stacks. Hence, in this approach, all the available spectral information was used without resorting to simplified vegetation indexes. Overall, classification performance was very high, with 98% sensitivity and 100% precision in a test set of 71 trees, 75% of which presented OQDS.

Moreover, the proposed method is computationally feasible. Indeed, processing times for both segmentation and classification were fast enough, amounting to about 6 s. This implies that this system is adequate for faster and less expensive monitoring of olive orchards than sampling in the field by agronomists and laboratory analysis.

As an alternative approach to segmentation, the authors are experimenting with the use of convolutional neural networks and have obtained promising results that will be illustrated in a future publication based on a larger dataset.

## Figures and Tables

**Table 1:** Spectral characteristics of the MicaSense RedEdge-M multispectral camera sensors (FWHM = full width at half maximum).

Sensor	Central Wavelength (nm)	Filter Bandwidth (FWHM) (nm)
Blue	475	20
Green	560	20
Red	668	10
Near-IR	840	40
Red-Edge	717	10

**Table 2:** Main mathematical symbols. The parameters of the algorithm are in bold.

Symbol	Description
$N_{r},\;N_{c}$	Number of pixel rows and pixel columns in each band of the multispectral reflectance stack
(r,c)	Row and column indexes of a pixel in a band, $r\;=\;1,\ldots ,N_{r}$ and $c=1,\ldots ,N_{c}$.
BLUE(r,c), GREEN(r,c), RED(r,c), NIR(r,c), REDEDGE(r,c)	Reflectances corresponding to pixel index (r,c) of the five bands.
K	Number of pixels in a given image that match with other images for 3D reconstruction with photogrammetry
P	Set of matched pixels
$\left(u_{i},v_{i}\right)$	Pixel indexes of matched pixels, $(u_{i},v_{i})\in P,\;i\;=\;1,\ldots ,K$
$\left(x_{i},y_{i},z_{i}\right)$	3D coordinates corresponding to matched pixels, i = 1,…,K
$e_{i}^{\prime }$	Standardized difference between $z_{i}$ and interpolating plane
$t_{H}^{\prime }=0$	Standardized elevation threshold
$s_{F}=50,\sigma_{F}=0.5,\;$ $m_{F}=50$	Parameters for Felsenszwalb’s oversegmentation, respectively: scale; standard deviation of Gaussian kernel for preprocessing of image; minimum component size.
$N_{F}$	Number of Felsenszwalb’s segments
$F_{j}$	Set of pixels indexes (r,c) in the jth Felsenszwalb’s segment, $j\;=\;1,\ldots ,N_{F}$. It is used to indicate that segment.
$a_{j}$	Number of pixels in $F_{j}$
$S_{j}^{\prime },S_{j}^{\prime \prime },S_{j}$	Results of the first, second, and final segment-classification method, respectively
$C_{NT},C_{T},C_{U}$	Classes defined for the segment-classification methods:$\;C_{NT}\;$(“*not part of a tree*”), $C_{T}$ (“*part of a tree*”) and $C_{U}$ (“*unknown*”)
$t_{area}^{r}=0.1$	Relative area threshold for the first segment-classification method
$t_{NIR}=0.15$	Mean NIR reflectance threshold for the first segment-classification method
$t_{NDVI}=0.5$	Mean NDVI reflectance threshold for the first segment-classification method
$p_{T,j}$	Probability of a segment $F_{j}$ of being in class $C_{T}$ calculated by the LDA classifier of the second segment-classification method
$t_{T}=0.3$	Probability threshold of $p_{T,j}$ for putting $F_{j}$ in class $C_{T}$
$C_{0},C_{1}$	Classes defined for health status classification of trees: $C_{0}$ (“*negative*”) when they are in good health status, and $C_{1}$ (“*positive*”) for bad health status
$X_{0},X_{1}$	Classes defined for pixels of negative and positive trees, respectively
p(u,v)	Probability that a pixel of coordinates (u,v) belongs to the class $X_{1}$ of pixels of positive trees
$r_{e}=4$	Radius in pixels of the disk for morphological binary erosion of segmented trees
L	Labeled image of connected components of segmented trees
T	Set of pixels of a given component in L, representing a segmented tree
M	Number of pixels in T
P	Set of probabilities associated with pixels in T, relevant to a given tree segment
$\left[p_{\left(1\right)},\ldots p_{\left(M\right)}\right]$	Sequence of probabilities calculated by the LDA classifier for pixels in a given set T, sorted from higher to lower probability
N=2	Number of highest-probability pixels used for tree classification
v	Mean probability value over N pixels for a given set T
t=0.8	Probability threshold for classifying a segmented tree into $C_{1}$

**Table 3:** Statistics of DSC for the training set.

# of Trees	71
Mean DSC	0.68
Std DSC	0.16
# of trees with DSC < 0.5	10
# of trees with DSC < 0.25	1
# of trees with DSC = 0	0
Min DSC	0.12

**Table 4:** Confusion matrix for the training set.

Ground Truth	Predicted Negative	Predicted Positive
Negative	15	4
Positive	0	52

**Table 5:** Statistics of DSC for the test set.

# of Trees	71
Mean DSC	0.66
Std DSC	0.21
# of trees with DSC < 0.5	16
# of trees with DSC < 0.25	4
# of trees with DSC = 0	0
Min DSC	0.02

**Table 6:** Confusion matrix for the test set.

Ground Truth	Predicted Negative	Predicted Positive
Negative	18	0
Positive	1	52
